# Poster Session II - A311 MENTAL HEALTH BURDEN OF PATIENTS WITH INFLAMMATORY BOWEL DISEASE AT CHUS SHERBROOKE IN THE AFTERMATH OF THE COVID-19 PANDEMIC

**DOI:** 10.1093/jcag/gwaf042.311

**Published:** 2026-02-13

**Authors:** O Harati, P Gaulin, K Hamel, M Malick, S Plamondon, M Delisle

**Affiliations:** Gastroenterology Residency Program, Sherbrooke University, Sherbrooke, QC, Canada; Gastroenterology Residency Program, Sherbrooke University, Sherbrooke, QC, Canada; Internal medicine department, Sherbrooke, QC, Canada; Department of Medicine, CHUS, Sherbrooke, QC, Canada; Department of Medicine, Gastroenterology Division, CHUS, Sherbrooke, QC, Canada; Department of Medicine, Gastroenterology Division, CHUS, Sherbrooke, QC, Canada

## Abstract

**Background:**

Patients with inflammatory bowel disease (IBD) have a high prevalence of mental health disorders which are associated with worse disease outcomes. Additionally, the global COVID-19 pandemic has led to an increased incidence of mental health challenges in the general population.

**Aims:**

This study aims to assess the prevalence of anxiety and depression in a cohort of patients with IBD following the COVID-19 pandemic.

**Methods:**

The prevalence of anxiety and depression was assessed in IBD outpatients, either in person or via virtual visits, using the validated Patient Health Questionnaire-9 (PHQ-9) and Generalized Anxiety Disorder-7 (GAD-7) scales. Patients’ demographics, disease characteristics, and treatment history were collected through retrospective chart reviews. Multivariable analysis was performed to identify factors associated with depression and anxiety.

**Results:**

A total of 199 patients (59.3% female, mean age 47) completed the questionnaires. Of these, 130 (65.3%) were treated with advanced therapies. 15.1% met the criteria for depression and/or anxiety. The prevalence of depression was 13% while anxiety was observed in 7.5% of patients. 11 patients (5.5%) met the criteria for both diagnoses, see *table 1*. A total of 41 patients (20.6%) were receiving treatment for depression or anxiety. Hospitalization since 2020 (32 patients) was the only factor significantly associated with both depression and anxiety (p = 0.0001; 95% CI: 2.48–12.92). Corticosteroid therapy (p = 0.063; 95% CI 0.94–4.74) and disease activity (p = 0.127; 95% CI 0.72–14.02) appeared to increase risk, while treatment changes (p = 0.626; 95% CI 0.55–2.66) were not associated with mental health symptoms. Most patients (77.9%) were satisfied with their gastroenterologist’s attention to mental health, and 68.8% wanted integration of mental health services into their IBD care.

**Conclusions:**

A substantial proportion of IBD patients in out cohort experience depression and anxiety, with recent hospitalization as a key risk factor. Most patients desire improved access to mental health resources, supporting the integration of these services into IBD clinics and post-hospitalization care.

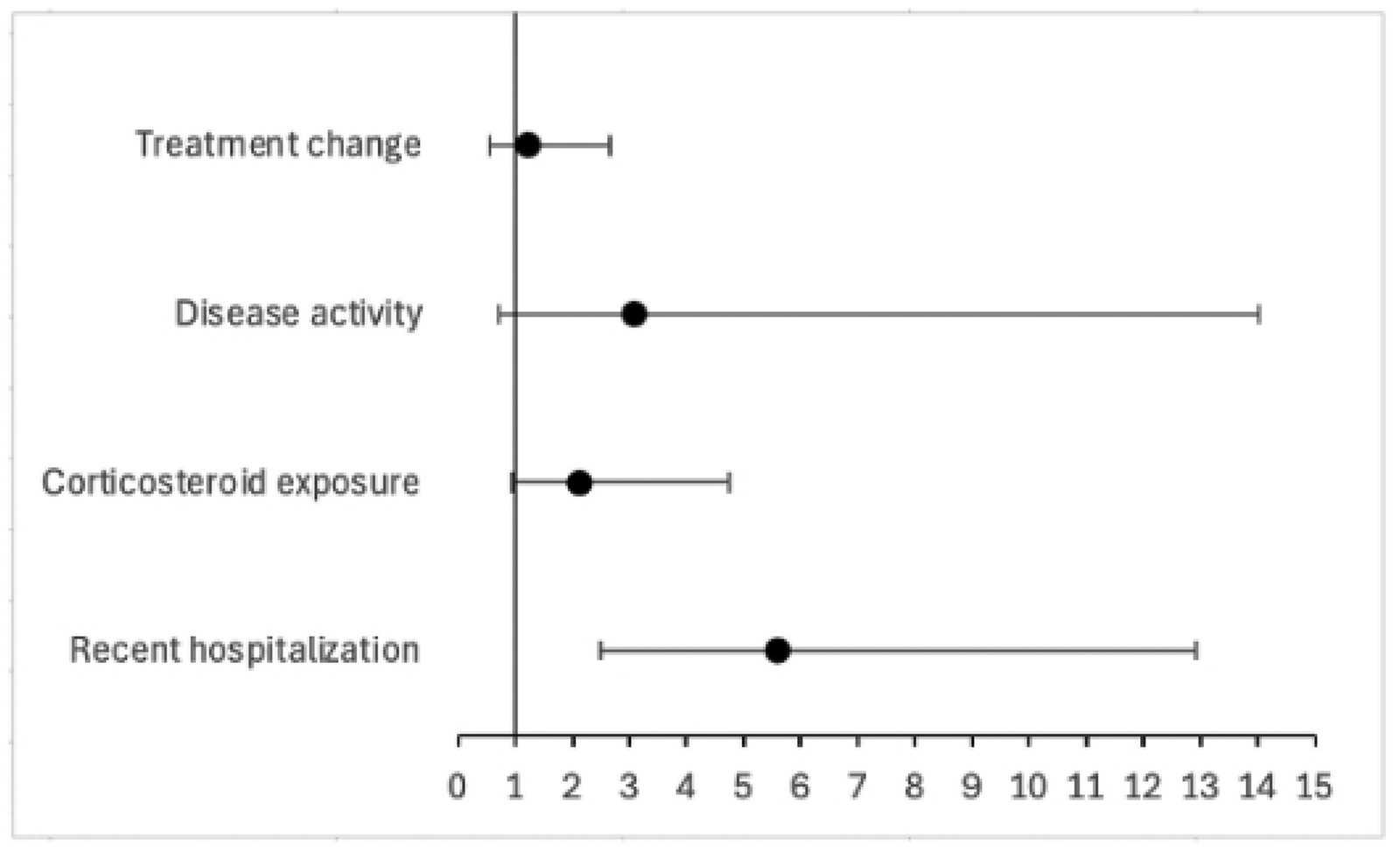

**Funding Agencies:**

None

**A311 Table 1:** Severity of mental health symptoms in patients with inflammatory bowel disease in our cohort

Mental Health Questionnaires	Overall (N = 199)
PHQ 9	N = 199
No depression	124 (62.3%)
Mild depression	49 (24.6%)
Moderate depression	23 (11.5%)
Severe depression	3 (1.5%)
GAD 7	N = 199
No anxiety	156 (78.4%)
Mild anxiety	28 (14.1%)
Moderate anxiety	10 (5.0%)
Severe anxiety	5 (2.5%)

